# Visuomotor adaptation needs a validation of prediction error by feedback error

**DOI:** 10.3389/fnhum.2014.00880

**Published:** 2014-11-04

**Authors:** Valérie Gaveau, Claude Prablanc, Damien Laurent, Yves Rossetti, Anne-Emmanuelle Priot

**Affiliations:** ^1^INSERM U1028, CNRS UMR5292, Lyon Neuroscience Research CenterBron, France; ^2^Université Claude Bernard Lyon 1Villeurbanne, France; ^3^Mouvement et Handicap, Hôpital Neurologique Pierre Wertheimer, Hospices Civils de LyonBron, France; ^4^Institut de Recherche Biomédicale des Armées (IRBA), Brétigny-sur-Orgecedex, France

**Keywords:** eye-hand coordination, visuomotor adaptation, prism adaptation, unawareness, prediction error, feedback error, internal model

## Abstract

The processes underlying short-term plasticity induced by visuomotor adaptation to a shifted visual field are still debated. Two main sources of error can induce motor adaptation: reaching feedback errors, which correspond to visually perceived discrepancies between hand and target positions, and errors between predicted and actual visual reafferences of the moving hand. These two sources of error are closely intertwined and difficult to disentangle, as both the target and the reaching limb are simultaneously visible. Accordingly, the goal of the present study was to clarify the relative contributions of these two types of errors during a pointing task under prism-displaced vision. In “terminal feedback error” condition, viewing of their hand by subjects was allowed only at movement end, simultaneously with viewing of the target. In “movement prediction error” condition, viewing of the hand was limited to movement duration, in the absence of any visual target, and error signals arose solely from comparisons between predicted and actual reafferences of the hand. In order to prevent intentional corrections of errors, a subthreshold, progressive stepwise increase in prism deviation was used, so that subjects remained unaware of the visual deviation applied in both conditions. An adaptive aftereffect was observed in the “terminal feedback error” condition only. As far as subjects remained unaware of the optical deviation and self-assigned pointing errors, prediction error alone was insufficient to induce adaptation. These results indicate a critical role of hand-to-target feedback error signals in visuomotor adaptation; consistent with recent neurophysiological findings, they suggest that a combination of feedback and prediction error signals is necessary for eliciting aftereffects. They also suggest that feedback error updates the prediction of reafferences when a visual perturbation is introduced gradually and cognitive factors are eliminated or strongly attenuated.

## INTRODUCTION

That sensorimotor adaptation can be induced by wearing prisms, which shift the visual field laterally, has been known since at least von Helmholtz (1910). When a subject wearing prisms is asked to point quickly to a near object, (s) he initially points to the prism-displaced image of the object, experiencing a pointing error. After tens of pointing attempts, the pointing error is gradually reduced to zero. When prisms are removed, the subject unexpectedly experiences a pointing error in the opposite direction to that induced by the prisms. This negative aftereffect persists after a few trials. This simple experiment provides one of the simplest illustrations of the short-term plasticity of the central nervous system (CNS), which allows it to adapt to changes in the relationships between visual inputs and corresponding motor outputs (for a review, see Redding et al., 2005).

While the existence of short-term sensorimotor plasticity is well established, the nature and origin of the error signals involved in eliciting the adaptation are still a matter of controversy. Three main sources of error have been suggested to induce adaptation: (1) a discrepancy between vision and proprioception of the hand (Craske and Crawshaw, 1974; Redding and Wallace, 1992a); (2) an inconsistency between predicted visual reafferences of the moving hand (derived from an efferent copy) and actual visual reafferences, as suggested by Held’s efference–reafference theory (Held and Hein, 1958) or by more modern versions of this theory introducing internal models (Miall and Wolpert, 1996; Kawato, 1999; Diedrichsen et al., 2005; Shadmehr et al., 2010); (3) a reaching feedback error, i.e., the simultaneous vision of the target and hand either during movement (Redding and Wallace, 1988) and/or at movement end (Harris, 1963; Welch and Abel, 1970; Kitazawa et al., 1995; Martin et al., 1996; Magescas and Prablanc, 2006). Welch and Abel (1970) coined the term “target pointing effect” to describe the observation that reaching to a prism-displaced visual target shows more adaptation than when reaching to no visual target. Under most conditions, these sources of error are closely intertwined. The first one, the discrepancy between vision and proprioception of one’s own limb, is known to produce a miscalibration of the visual reference frame or/and of the hand proprioception (Redding et al., 2005; Cressman and Henriques, 2010; Scarpina et al., in press). In the following, to avoid confusion, we consider adaptation paradigms which minimize the influence of sensory (visual and proprioceptive) adaptive rearrangements.

According to the influential theory of Held and Hein (1958), Held (1961), Held and Freedman (1963), visuomotor adaptation uses the comparison between predicted (the efference copy) and reafferent visual signals. Efference copy refers to the copy of the motor output resulting from a motor command (or sometimes, to the copy of this motor command) while reafferent visual signal refers to the visual afference resulting from this motor command, i.e., vision of the actively moving hand. Visuomotor adaptation results from the progressive decrease of the conflict between the efference copy and reafferent signals. A similar theory was further formalized by introducing the concept of forward internal model, i.e., an instantaneous copy of the state (position and velocity) of the limb representing a prediction of the visual reafferences (Miall et al., 1993; Wolpert et al., 1995; Miall and Wolpert, 1996; for a review, see Wolpert, 1997; Wolpert et al., 1998; Kawato, 1999). Adaptation is induced by the conflict between the forward internal model output (efference copy) and the corresponding visual reafferences that we referred to as the prediction error as classically considered by Held (1961), Miall and Wolpert (1996), or Tseng et al. (2007). While many studies have suggested that prediction-error processing is the main source of adaptation (Held, 1961; Diedrichsen et al., 2005; Mazzoni and Krakauer, 2006; Tseng et al., 2007; Shadmehr et al., 2010; Taylor and Ivry, 2011; Izawa et al., 2012), the respective roles of feedback error and prediction error have not been completely elucidated, because some feedback error was usually present, or uncertain, in these studies. We consider feedback error *per se* as the static or dynamic visual error between the goal-directed target (or any physical or short-term memorized visual landmarks) and the pointing hand. Such an isolation of pure feedback error condition without prediction error has been obtained using a target-jump paradigm by Magescas and Prablanc (2006), Cameron et al. (2010) and Laurent et al. (2011). These authors showed that a strong and robust reaching adaptation could be elicited by a terminal feedback error signal, in the absence of any conflict between predicted and actual reafferences. This experiment, similar to the classical saccadic-adaptation paradigm in eye-movement control (for a review, see Hopp and Fuchs, 2004), established that a robust adaptation could be elicited by a change in the inverse model converting the goal of an action into motor commands.

The aim of the present study was to determine the relative contributions of retinal feedback error and non-feedback prediction error in prism-induced reaching adaptation as defined above, when subjects are unaware of the prism-induced conflict. Two distinct experiments were performed in order to separate the two error signals, by allowing the opening or closing of the external feedback loop (vision of subject’s hand) at controlled times of the execution of a pointing movement. In a first condition, hereafter referred to as the “terminal feedback error” condition, vision of the pointing hand was allowed at movement end only. The only available source of error was the simultaneous vision of the prism-displaced hand and target at movement end. In a second condition, hereafter referred to as “movement prediction error,” vision of the pointing hand during exposure was limited to the duration of a self-initiated movement performed under a black homogenous background, in the absence of any visual target or landmark. The error signal arose solely from the comparison between the predicted visual feedback from the moving hand and the visual percept of the actual hand position, without any other cue. Consistent with the results from the double-step adaptation paradigm of Magescas and Prablanc (2006), which highlight the role of terminal feedback error, we expected that the “movement prediction error” condition would generate a lower level of adaptation than the “terminal feedback error” condition owing to a lower accuracy of the discrepancy between the seen hand and its visual prediction than those of a physical retinal error.

## MATERIALS AND METHODS

### SUBJECTS

Ten naive right-handed subjects (five female and five male, mean age = 19.8 years, SD = 0.7 years) took part in the experiment. All subjects had normal or corrected-to normal vision, and no history of neurological or psychiatric disorders. They all provided informed consent prior to participation. The experiment was conducted in accordance with the Declaration of Helsinki and under the terms of local legislation.

### APPARATUS

**FIGURE 1:**
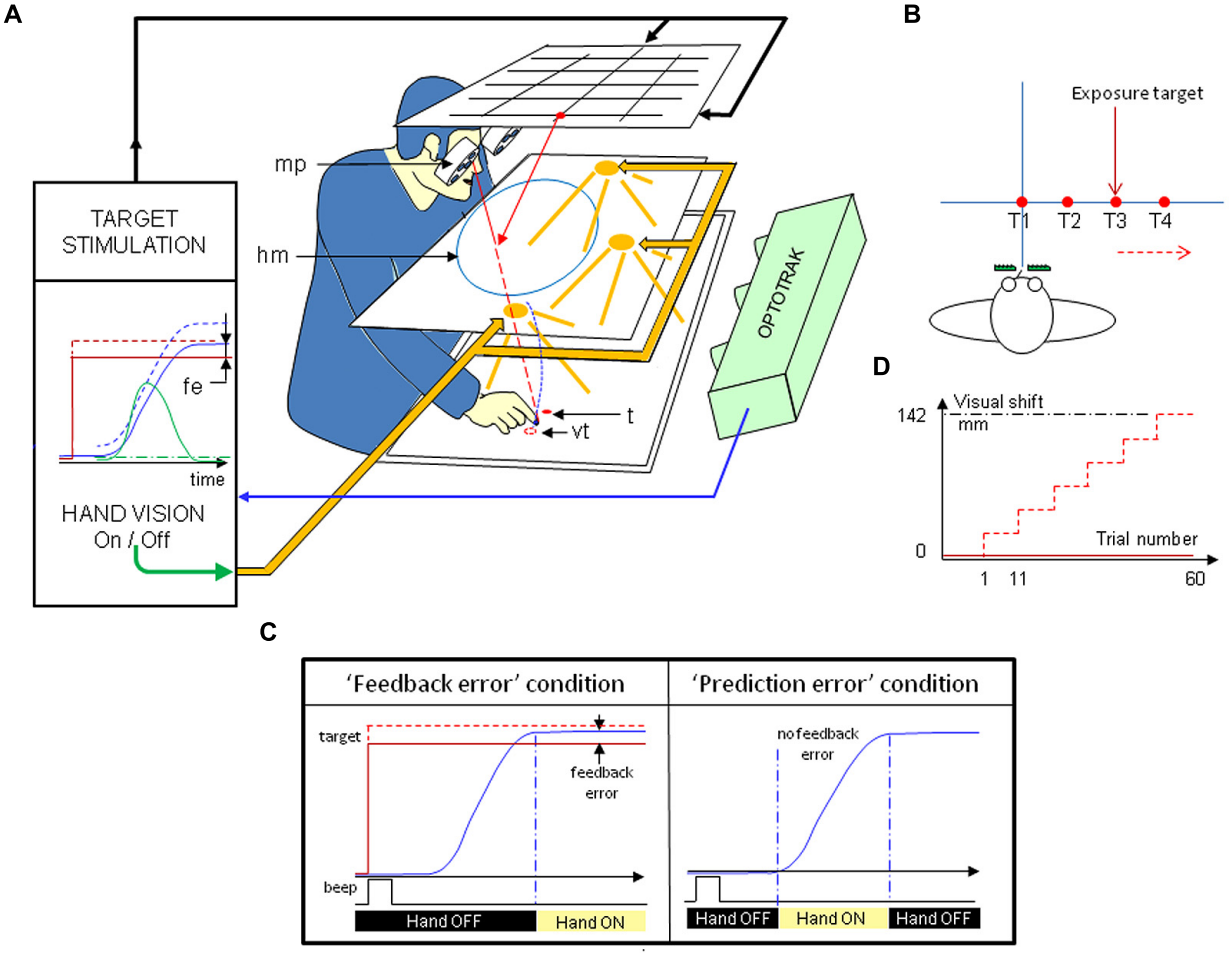
**(A)** Experimental setup (modified from Prablanc et al., 1979). Targets are seen through a half-reflecting mirror (*hm*) and appear to be placed on the pointing surface. The target (*t*), mirror image of the light-emitting diodes on the upper stimulation plane is shown as a filled red circle. The prism-displaced image of the target (*vt*) is shown as an open red circle. The pointing hand is only visible when the volume between the mirror and the pointing surface is lit. An infrared-emitting diode is attached to the right index fingertip, the position of which is recorded. A set of either neutral or right deviating Fresnel prisms is placed in front of the eyes, uncovering a large visual field (around ± 30°). Prisms are mounted on a motorized disk (*mp*), which allows quick switching, from zero to any prism deviation. Vision of the target and limb was monitored, and this information was used to open or close the external feedback loop (vision of subject’s hand), and to adjust the binocular prism settings through fast step-motors. Opening or closing the feedback loop was determined by the crossing of a velocity threshold. Red solid line: physical target; red dotted line: seen target; blue solid line: physical hand; blue dashed line: seen hand; green solid line: hand velocity. **(B)** Layout of the targets. The four targets (T1, T2, T3, T4) used in the pre- and post-tests were located along a fronto-parallel line, at, respectively, 0, 100, 200, 300 mm right of the midline. The four targets are used during pre- and post-tests, while T3 is used during “terminal feedback error” condition exposure and no target is used during “movement prediction error” condition exposure. **(C)** Prism-induced visual displacement during exposure. The rightward prism deviation of a single target T3 was incrementally shifted from 4 to 25 diopters (resulting in a 142-mm rightward displacement), every block of 10 trials. **(D)** Real-time control of target LEDs and vision of the limb during the exposure period. Please see text for details. Solid red line: physical target; dotted red line: seen target; solid blue line: hand; vertical dashed and dotted blue line: movement onset and offset.

The visual stimulation consisted in red light-emitting diodes (LED) placed on a plane located horizontally above the subject’s head (**Figure 1A**). As subjects observed the targets through a half-reflecting mirror, the targets appeared on a horizontal table on which the subjects were pointing. Because the target was a virtual image, finger-to-target masking could not influence the results. The (virtual) images of T1, T2, T3, and T4 targets were located 0 to 30 cm rightward from the subject’s sagittal axis in 10-cm increments, respectively, along a fronto-parallel line 57-cm away from the subject’s eyes (**Figure 1B**).

The subject sat on a medical chair in front of the pointing surface. The pointing surface was a black flat and matte surface without a visual frame of reference or any other distinctive landmark, and extending across the entire visual field. A non-visible tactile landmark served as a starting point for pointing movement. It was placed 20 cm away from the subject along the sagittal axis. Direct vision of the pointing hand through the half-reflecting mirror could be prevented by turning off a set of power white LEDs placed between the mirror and the pointing table. This electronically controlled optical device allowed the subject to view his/her upper limb and hand over the black background during movement, and it prevented other spatial cues in the absence of a visual target. It also made it possible to mask the view of the hand prior to, and during, the movement to the visual target, and to let the hand be visible after the end of the movement only. Therefore, this apparatus made it possible to dissociate non-retinal dynamic errors resulting from discrepancies between the expected and actual visual reafferences of the moving limb (“movement prediction error”), and physical hand-to-target error signals resulting from the simultaneous vision of the target and limb (“terminal feedback error”). Care was also taken to avoid dynamic retinal errors, such as moving hand-to-landmark visual errors, by providing a black fully homogenous background which allowed prediction errors only. Dynamic retinal errors are the instantaneous hand-to-target signal which gives both velocity and position error signals relative to target. Schween et al. (2014) observed the capability of these dynamic signals to drive some adaptive process.

During the exposure phase, the subject experienced prism viewing. The prism device was composed of seven pairs of Fresnel prism sheets (Press-On 3 M) mounted on two computer-controlled motorized disks. This optical arrangement deviated the line of sight of each eye rightward, with a large circular visual field that allowed subjects to see the forelimb and hand during pointing. The amount of prism deviation ranged from 0 to about 14° (corresponding to 0, 4, 8, 12, 15, 20, and 25 diopters). Notice that seeing through Fresnel prism introduces vertical stripes. In order to prevent some knowledge of context, the zero-diopter prism consisted of a neutral transparent sheet with stripes. Small and progressive (computer-controlled) prism increments in exposure avoided awareness of errors and strategic correction (Magescas and Prablanc, 2006; Michel et al., 2007).

An infrared emitting diode was attached to the right index fingertip, the position (x, y horizontal components with a 0.1-mm accuracy) of which was recorded at 100 Hz with an Optotrak 3020, Northern Digital Inc. Real-time monitoring of the target LEDs and of limb vision was performed using an ADWIN (Keithley-Metrabyte) system. This was used to open or close the external feedback loop (vision of subject’s hand), and to control the targets and the fast step-motors that controlled the binocular prism settings. Online detection of hand pointing movement was determined by a fixed 20-mm displacement and a 80-mm/sec velocity threshold, using a two-point central difference algorithm with a 10-ms binwidth (Magescas and Prablanc, 2006).

Note that a discrepancy between seen and felt hand positions is inherent to prism exposure and that such a discrepancy can induce sensory (visual and proprioceptive) adaptation (Harris, 1963; Redding et al., 2005; Priot et al., 2011). Several studies have shown that a minimum movement duration of 1 s or more is required for prism adaptation induced by visual-proprioceptive conflict (for a review, see Redding and Wallace, 1992b). To reduce the efficiency of the visual-proprioceptive error signal, the prism-displaced vision of the hand was strictly limited to the movement duration in the “movement prediction error” condition, and to 0.5–1 s after movement end in the “terminal feedback error” condition. Thus, although a visual-proprioceptive mismatch was present in both experiments, its influence was minimized by limiting the duration of the inter-sensory mismatch. We aimed at mitigating any static visual-to-proprioceptive conflict, in order to measure the motor component of visuomotor adaptation. In addition, as initial vision of the hand (either neutral or altered) is known to influence movement planning (Rossetti et al., 1994, 1995; Desmurget et al., 1995), the movement was always initiated without vision of the hand in order to avoid confounding effects. As both conditions involved natural free-hand pointing without an artificial interface, with similar kinematics and movement durations, the difference between aftereffects should reflect the influence of each type of adaptation. In addition, the subject’s lack of awareness of a conflict, and his/her ability to see his/her hand naturally (as opposed to seeing a cursor or a handle) allowed us to study adaptation, rather than learning (Redding et al., 2005).

The “terminal feedback error” condition was not restricted to a pure retinal error. It also included a small predictive component. Indeed, starting on the first trial within a block of ten, the subject expected his pointing to be on the target and not statistically displaced on the right. However, this predictive component was not consciously perceived as an external manipulation, because the prism increment (corresponding approximately to a 2-cm displacement of the target) was close to the variability of open-loop pointing (mean standard deviation for individual participants = 15.7 mm), when the visual reafferences were absent.

### PROCEDURE

Each subject took part in the two experimental conditions described above. An ABBA design with a two-week delay between the experiments was used to mitigate order effects. A standard experimental paradigm, including three blocked sessions (pre-test, exposure to prisms, and post-test) was used. Pre- and post-tests were the same for both conditions. All movements performed during either the test or the exposure sessions were carried out with a natural parabolic path at a natural and comfortable speed. Fixation points prior to movements were not used during either the tests or the exposure phases, so that subjects would have no other spatial cue besides body-centered target information when the target was lit under an otherwise dark background.

#### Pre- and post-tests

Pre- and post-tests were identical. At the beginning of each trial, the subject was in total darkness and did not see his/her hand or the table. The prism was in the neutral position (0 diopter). The subject heard a 10-ms beep indicating that he/she had to place his/her right index at a starting position defined by a tactile cue on the pointing table. When the index was within ± 10 mm around the starting position, a second 100-ms beep occurred simultaneously with the lighting of one the four targets instructing the subject to point to the target without vision of his/her hand. To minimize error signals arising from discrepancies between his felt index and seen target position, the target was turned off as soon as the subject pointed to it (as determined by the hand-velocity threshold). About 500 ms after the target was turned off, the subject was instructed to move back onto the tactile starting position and the next trial started. Pre- and post-tests were composed of 10 blocks of the four targets (T1 to T4) presented in a pseudo-random order.

#### Exposure

In the first condition named “terminal feedback error” exposure, the only available source of error was the simultaneous vision of the hand and target at movement end, without visual feedback of the hand during movement (**Figure 1C**). At the beginning of each trial, the subject was in total darkness and did not see his/her hand or the table. The subject heard a 10-ms beep indicating he/she had to place his/her right index at a starting position defined by a tactile cue on the pointing table. A second 60-ms beep signaled that the index was within ± 10 mm around the starting position. The binocular prisms were initially set at 4 diopters. 500 ms after the beep, the T3 target (20-cm right) was turned on. The subject had to point to the visual target without vision of his/her hand. At the end of the pointing movement (based on hand-velocity threshold), the high-power white LED illuminated the pointing table, allowing full forelimb and hand terminal feedback error, i.e., the simultaneous vision of the target and hand at movement end for a 0.5–1 s duration. The target and illumination of the hand were then simultaneously turned off, the subject was instructed to move back onto the tactile starting position, and the next trial started. The return movement was thus carried out in the dark. The rightward prism deviation was incrementally increased to 4, 8, 12, 15, 20, and 25 diopters every block of 10 trials (**Figure 1D**), resulting in 60 trials with a final prism deviation of 0.25 × 570 = 142 mm.

In the “movement prediction error” condition, the vision of the hand was limited to the duration of motion, in the absence of visual target (**Figure 1C**). The beginning of each trial was identical to those described for “terminal feedback error” exposure, except that after the beep signaling that the index was within ± 10 mm around the starting position, the subject had to point ahead of the shoulder at a distance corresponding to that of the T3 target (experienced during pre-test), without vision of his/her hand. No target was lit, but the intended pointing position was roughly at the level of the parasagittal plane of the shoulder, i.e., nearly corresponding to the T3 target. Direct vision of the hand was turned on at movement onset by turning on the high-power white LED on the pointing table and was turned off at the end of movement in order to prevent the perception of a final discrepancy between seen and felt limb. Just after his/her limb became invisible, the subject was instructed to move back onto the tactile landmark starting position in the dark and next trial was launched. As in the “terminal feedback error” condition, the rightward prism deviation was incrementally shifted from 4 to 8, 12, 15, 20 and 25 diopters, every block of 10 trials. The pointing instruction was repeated at each prism increment in order to keep subject’s focus on his/her task.

For both “terminal feedback error” and “movement prediction error” conditions, prism increments were computer-controlled, so that there was no break during the exposure, which might have allowed the subjects to form cognitive strategies about prism increments. In addition, to avoid contextual influences, the striated lines introduced by the Fresnel prisms were also reproduced during the (no prism) pre- and post-tests.

Pointing movements during exposure were likely to be similar across the two conditions. In the “terminal feedback error” condition, the visual target was close to the shoulder parasagittal plane whereas in the “movement prediction error” condition, there was no target and the subject was instructed to point along the parasagittal plane of the shoulder, at a distance corresponding to that of the target during the pre-test. Consequently, movement path and durations during the exposure were similar across the two conditions.

### DATA ANALYSIS

Pointing errors were defined as the difference between the final position of the fingertip and the position of the target along the *x* axis in the pre- and post-tests. For the “terminal feedback error” condition, pointing errors during exposure were defined as the difference between the fingertip final position and T3-target position along the *x* axis. For the “movement prediction error” condition, there was no physical error during the exposure, as no target was present. The average pointing position across the first ten pointing was taken as a reference for each subject, and was used to compute an arbitrary pointing error along the *x* axis. For all pointing, leftward errors were assigned a negative value and rightward errors a positive value. We also computed the mean variable pointing error during the pre-tests, which was defined as the standard deviation of pointing, for each target and each subject. A mean variable pointing error was also computed during exposure, and defined as the standard deviation of pointing, for each prism deviation and each subject. Only the six last pointing were taken into account, corresponding to stabilized pointing after twice the decay time-constant.

Prior to averaging the data, possible temporal drifts in pointing behavior across trials during the pre-test and exposure trials were checked using Spearman correlation, with pointing error as a dependent variable and trial number as an independent variable.

In order to ensure that the initial pre-test performance (taking into account both absolute and variable errors) was not different between the two conditions, and not influenced by testing order, a repeated-measure ANOVA was performed using the mean pointing error, or mean pointing variable error, during the pre-test as a dependent variable, the condition as a within-subject two-level factor (prediction, feedback), the target position as a within-subject four-level factor (T1 to T4), and the condition test order as a between-subject two-level factor (prediction error followed by feedback error, feedback error followed by prediction error).

Changes in pointing error during exposure were quantified for each prism increment by fitting a function of the form *errx* = x_lim_+ *ae*^-(^*^i^*^-1)/^*^nc^*, with *a* > 0, *b* > 0, and *b* < 1, where *errx* denotes the error along the *x* axis, *x_lim_* is the asymptotic value of the error, *a* denotes the total amplitude decay of the error, *i* denotes the trial number, and *nc* denotes a decay time-constant.

In order to compare the accuracy of the two error signals, a repeated-measure analysis of variance (ANOVA) was performed using the mean pointing variable error during exposure as a dependent variable, the condition as a within-subject two-level factor (prediction, feedback) and the prism deviation as a within-subject six-level factor (P4 to P25).

In order to test for the presence of aftereffects, pointing-error means were computed for each subject and were compared using a repeated-measure ANOVA with session as a two-level factor (pre-test, post-test) and the target as four-level factor (T1 to T4). This analysis was performed for each condition (prediction and feedback) separately. Target position was included as a factor to analyze the generalization of adaptation to untrained directions. For each subject and each target, a mean pointing aftereffect was computed by taking the signed difference between the mean values across the 10 random repetitions of the pre-test and post-test pointing errors, or the signed difference between the pointing distance for the *n^th^* repetition and the mean values across the 10 random repetitions of the pre-test (as no temporal trend were observed during pre-test).

Although prism adaptation retention is usually quite strong, post-test effects have been found to decay slightly over time. Here, the temporal decay of the aftereffects as a function of target position was analyzed using an ANCOVA with the aftereffect as a dependent variable, the target as a four-level factor (T1 to T4), and the repetition number as a continuous factor.

A paired *t*-test was performed to compare the duration of pointing movement between the “terminal feedback error” and “movement prediction error” conditions, based upon the velocity threshold. Because of missing data for one subject, this test was run on eight subjects only.

## RESULTS

### PRE-ANALYSIS

The data were pre-analyzed to identify subjects who correctly performed the task during the exposure period. One subject was identified as an outlier because his mean z-score for pointing error during the “terminal feedback error” condition was equal to –6. This subject’s data were excluded from the analyses. This reduced the sample size from 10 to 9 subjects.

Preliminary analyses on pre-test data showed no difference between conditions (“feedback” vs. “prediction”) for the mean pointing (constant) error [*F*(1,7) = 2.16; *p* = 0.19] or the mean variable error [*F*(1,7) = 1.64; *p* = 0.24], and no effect of testing order of the conditions for mean pointing error [*F*(1,7) = 0.77; *p* = 0.41] or mean variable error [*F*(1,7) = 0.016; *p* = 0.9]. A statistically significant effect of target position was observed, with a trend toward overshooting that declined with eccentricity [*F*(3,21) = 7.07; *p* < 0.002] and increased variability for the most eccentric target (T4) [*F*(3,21) = 5.24; *p* < 0.01].

No significant correlation was found between pointing errors and trial number during pre-test of “terminal feedback error” and “movement prediction error” conditions (feedback: Spearman ρ = 0.07, *p* = 0.2; prediction: ρ = 0.11, *p* = 0.051), indicating stable pointing performance over trials.

The mean duration of pointing movement was 489 ms (SE = 21 ms) in the “terminal feedback error” condition and 476 ms (SE = 24 ms) in the “movement prediction error” condition. These two values did not differ significantly [*t*(7) = 0.46, *p* = 0.66].

### EVOLUTION OF POINTING ERRORS DURING EXPOSURE

**FIGURE 2:**
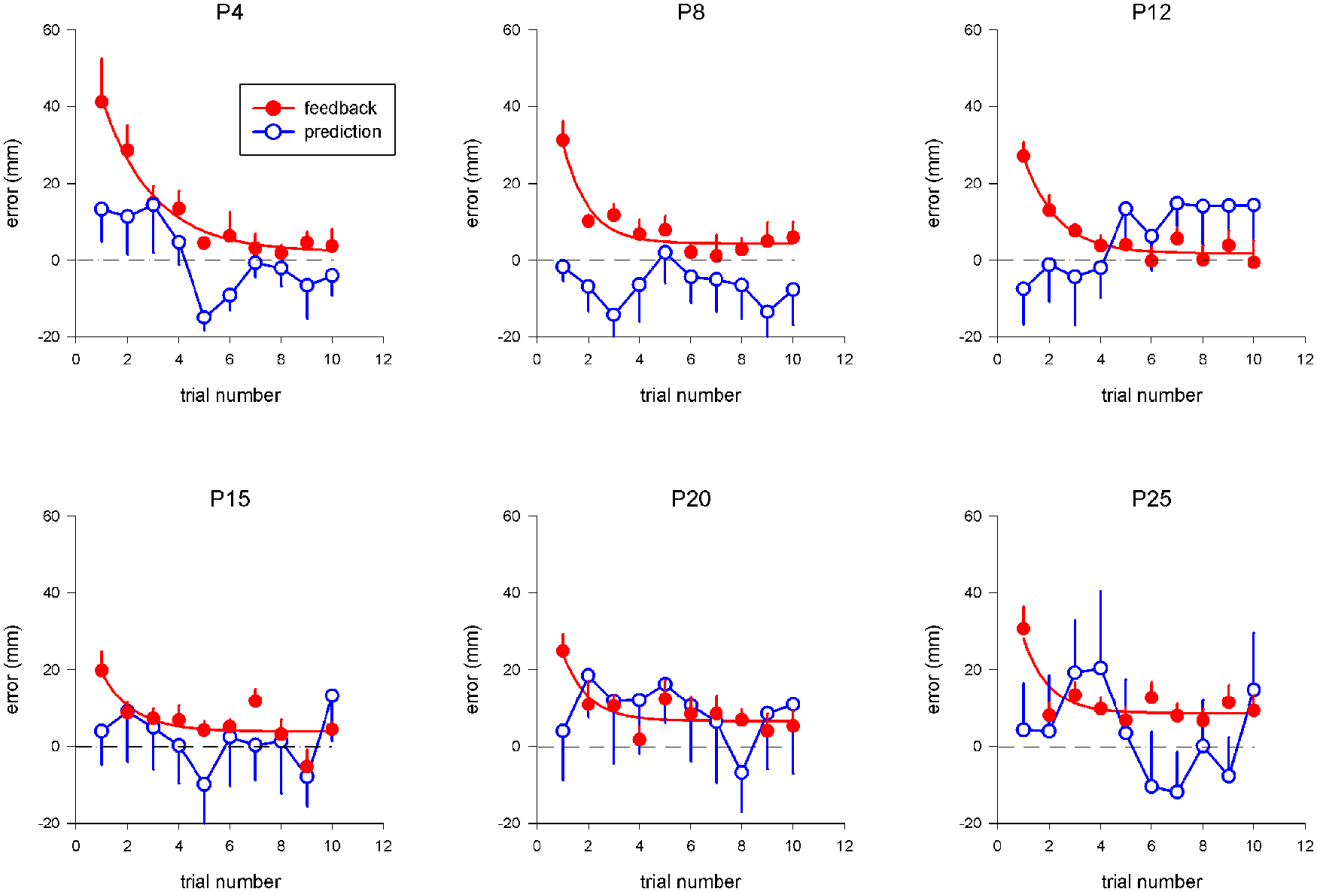
**Pointing error as a function of trial number and strength of prism deviation during exposure.** Each point represents the mean pointing error across all subjects (along the vertical axis) for a given trial number (along the horizontal axis), for a given prism deviation (P4 – P25), in a given condition (“terminal feedback error”: red filled circles; “movement prediction error”: blue open circles). For the “terminal feedback error” condition, pointing errors during exposure were defined as the difference between the fingertip final position and T3-target position along the *x* axis. For the “movement prediction error” condition, the average pointing position across the first ten pointing was taken as a reference for each subject, instead of T3-target. SE are indicated by vertical bars. For the “terminal feedback error” condition, exponential fits to the data are shown (red curves).

**Figure 2** shows pointing error as a function of trial number and prism displacement during exposure for the two tested conditions. For the “terminal feedback error” condition, a trend for pointing error to decrease during exposure was observed, and was well captured by an exponential-decay function fitted to the data; the best-fit parameters of this function are listed in **Table 1**. The asymptotic limit, *x_lim_*, increased linearly with prism deviation (*R* = 0.86; *p* < 0.03) up to 8.6 mm, after which it remained approximately constant, indicating a saturation of the adaptive process. In addition, the decay-constant decreased from 2 to 1 (trial) as a function of prism deviation.

**Table 1:** Parameters of the best-fitting exponential-decay function through mean pointing errors versus trial number in the “terminal feedback error” condition, with associated *R.*

Prism deviation (PD)	*x_lim_* (mm)	*a* (mm)	1/*nc*	*nc* (trial)	First trial error (mm)	*R*
4	2.1	39.9*	0.501*	2	42	0.99
8	4.3*	26.1*	1*	1	30.4	0.95
12	1.7	25.3*	0.777*	1.29	27	0.97
15	3.8	15.3*	0.804	1.24	19.2	0.77
20	6.6*	17.6*	1*	1	24.1	0.88
25	8.6*	19.5*	1*	1	28.1	0.88

*The *first trial error* represents the sum of the asymptotic value *x_lim_* and of the total amplitude decay *a*. Asterisks indicate one-tailed *p* values < 0.05 (calculated for xlim, a and 1/nc).*

For the “movement prediction error” condition, no systematic trend was observed during exposure (**Figure 2**), and the experience of conflict between intended hand-pointing movements and actual visual reafferences did not influence results on subsequent trials, even for large prism deviations—up to 25 diopters (14°), which correspond to lateral displacements of 142 mm of the seen hand position relative to the actual physical position.

A repeated-measure ANOVA showed a significant effect of condition on the mean variable pointing errors [*F*(1,8) = 9.28; *p* < 0.02], toward a larger variable error for “movement prediction error” condition (12.8 ± 1.9 mm for “movement prediction error” condition and 9 ± 1.9 mm for “terminal feedback error” condition).

### “TERMINAL FEEDBACK ERROR” VS. “MOVEMENT PREDICTION ERROR” AFTEREFFECTS

**FIGURE 3:**
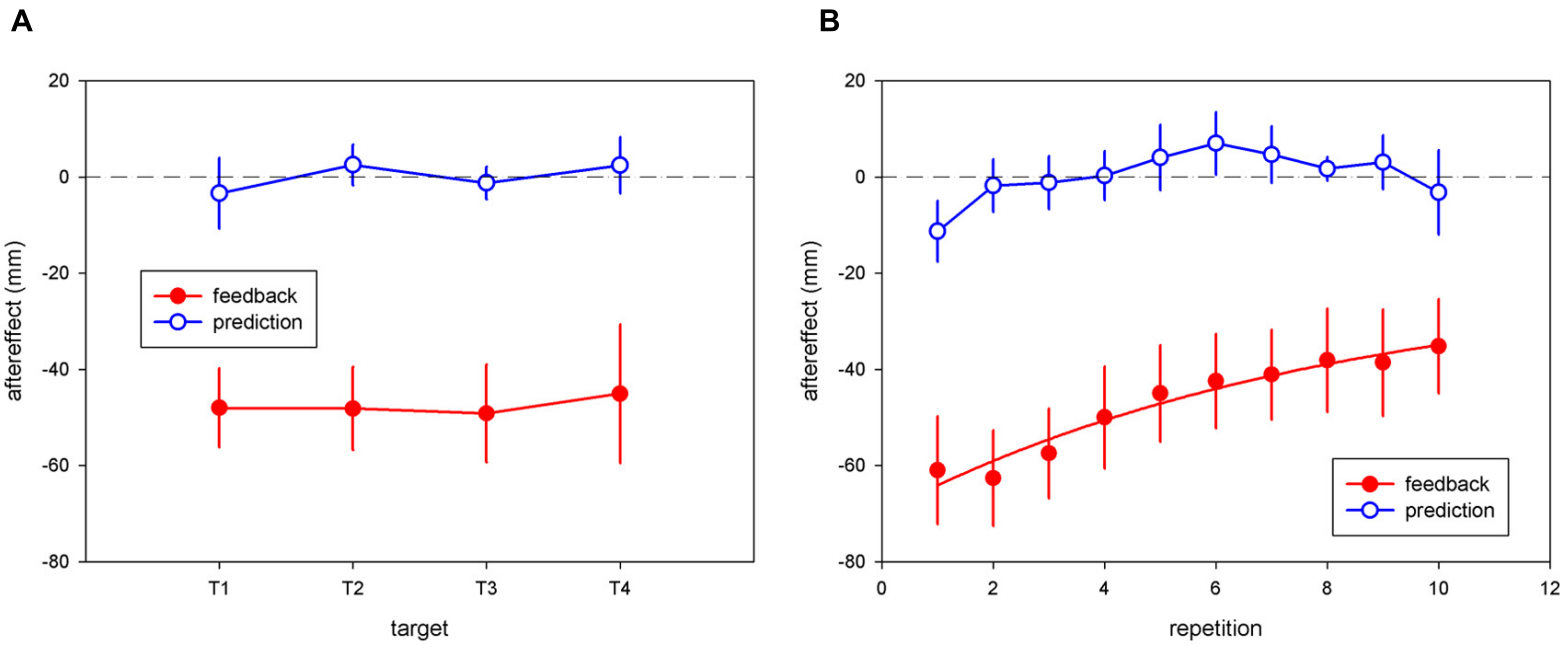
**(A)** Pointing aftereffects as a function of target for the “terminal feedback error” and “movement prediction error” conditions. Each point represents the mean aftereffect across all subjects during the “terminal feedback error” condition (filled red circles) or the “movement prediction error” condition (open blue circles). Standard errors are indicated by vertical bars. **(B)** Pointing aftereffects as a function of repetition number in the “terminal feedback error” and “movement prediction error” conditions. Each point represents the mean aftereffect across all subjects during the “terminal feedback error” condition (filled red circles) or the “movement prediction error” condition (open blue circles). Error bars show ± 1 SE of the mean across subjects for each condition. For the “terminal feedback error” condition, the best-fitting exponential-decay curve is shown.

Repeated-measure ANOVA on the mean pointing errors showed a significant effect of session for the “terminal feedback error” condition [*F*(1,8) = 23.18; *p* < 0.005] but not for the “movement prediction error” condition [*F*(1,8) = 001; *p* = 0.98]. There was no significant interaction between the session and target factors [*F*(3,24) = 0.14; *p* = 0.93] for the “terminal feedback error” condition, consistent with homogenous transfer of adaptation to untrained target locations (see **Figure 3A**). *T*-tests showed a significant aftereffect for each target: 48 ± 8.2 mm for T1 [*t*(8) = 5.83, *p* < 0.0005]; 48.1 ± 8.7 mm for T2 [*t*(8) = 5.55, *p* < 0.0001]; 49.1 ± 10.2 mm for T3 [*t*(8) = 4.8, *p* < 0.005]; 45 ± 14.5 mm for T4 [*t*(8) = 3.11, *p* < 0.05]. The average aftereffect, 47.6 mm, represented 33.5% of the 25-diopter (142 mm) deviation. No significant aftereffects were found for any of the target positions in the “movement prediction error” condition (minimum *p* > 0.56).

An ANCOVA on aftereffects for the “terminal feedback error” condition showed a significant effect of repetition number [*F*(1,32) = 75.08; *p* < 0.0001], but no effect of target [*F*(3,32) = 1.48; *p* = 0.24], and no interaction between target and repetition number [*F*(3,32) = 1.55; *p* = 0.22], consistent with a similar decline in aftereffects over time for all targets. Mean aftereffects (averaged across the four targets) decayed across trials (ρ = 0.98, *p* < 0.00001). The mean aftereffect on the first repetition, 61 mm, represented nearly 43% of the 25-diopter prism displacement applied on the last exposure trial (**Figure 3B**).

## DISCUSSION

This study investigated adaptation induced by prism exposure in two conditions: a “terminal feedback error” condition and a “movement prediction error” condition. Adaptive aftereffects were observed in the former condition only. The aftereffect, a shift of the movement in the direction opposite to that induced by the prism, was observed for all tested target eccentricities. For the final, 25-diopter (14°) deviation, subjects exhibited a 33.5% (of prism deviation) pointing aftereffect—43% (about 61 mm) when considering only the first few trials. This result is consistent with previous findings. For example, a previous study measured shifts of 40–80% (of the prismatic deviation) after about fifty trials of prismatic exposure, with either full vision of the hand and target during pointing, or terminal feedback error at movement end (Redding and Wallace, 1996). Unlike for the “terminal feedback error,” for the “movement prediction error” condition, no statistically significant aftereffect was observed. This results stands in contrast to those of the only previous study (to our knowledge) of prism adaptation that tried to dissociate these two error components. This study, which was performed by Redding and Wallace (1988), found an aftereffect of 25% following a 20-PD prism exposure with vision of the hand during, and after the end of, movement, without target. An important methodological difference between this previous study and the current one is that, in the former, two lateral vertical lines were visible on the background. It is likely that the two lines acted as landmarks, which could provide subjects with an indirect, dynamic feedback error (between the seen hand and the lines). This explanation, if it is correct, would be consistent with our hypothesis that hand-to-target or hand-to-landmark (continuous or terminal) feedback error signals play a key role in the adaptive process.

### GENERALIZATION OF ADAPTATION

The aftereffect generalized to unexposed targets in the “terminal feedback error” condition. This generalization more likely reflects an adaptive process which is context independent (Bedford, 1989; Vetter et al., 1999). Conversely, motor-learning process, as the latter type of effect is usually context-dependent and does not or little generalize (Cothros et al., 2006; Mattar and Ostry, 2007).

Any unaware learning during the last, 25-diopter exposure block, associating the prism-displaced image of the T3 target used during the exposure (140 mm rightward) with the pointing on T3, should have generated a maximum aftereffect for a target beyond T4, with a decreasing gradient for less eccentric targets (T4 to T1). By contrast, the aftereffect observed in the “terminal feedback error” condition generalized to untrained target locations. There is some confusion in the literature between adaptation and skill learning. On the one hand the prism adaptation literature (Redding et al., 2005) has been traditionally very careful in distinguishing between strategic compensation and “true adaptation” (Weiner et al., 1983). By contrast, the more recent literature on force-field and visual rotation skill learning tends to use both terms indistinctly, although Mazzoni and Krakauer (2006) clearly dissociate implicit adaptation and explicit strategy during visuomotor rotation. Compensation of initial errors during exposure can be achieved by either process, but true adaptation must be objectified by the measure of aftereffects (Weiner et al., 1983; Redding et al., 2005). A reduction of errors without aftereffect implies that the compensation has been achieved through strategic rather than adaptive mechanisms (Weiner et al., 1983; Pisella et al., 2004). The definition of aftereffects is another source of confusion between the two fields. While the prism literature has kept focus on assessing aftereffects in conditions departing from the exposure conditions (i.e., explicitly removing the glasses, unexposed target, different pointing speed, as described in Redding et al., 2005), the force-field or rotated feedback literature is keeping the subject in the training device to measure aftereffects (e.g., Herzfeld et al., 2014). Crucially, subjects exposed to prisms exhibit robust aftereffects after explicitly removing the goggles, while subjects exposed to a force-field exhibit little or no aftereffect when they are tested outside the apparatus (e.g., Cothros et al., 2006). Strategic compensation and true adaptation are not always easy to tease apart, and O’Shea et al. (2014) showed that they may coexist within a single reaching movement performed during prism exposure, yet distinctly leading to aftereffects or not. In addition, although they share many common properties, adaptation and skill learning are subtended by partially distinct processes. In force-field paradigms, it has been suggested that their differences reflect adaptive self-calibration of motor control versus learning the behavior of an external object or tool (Lackner and Dizio, 2005; Cothros et al., 2006).

### DISENTANGLING FEEDBACK AND PREDICTION IN ADAPTATION

The lack of an adaptation aftereffect for all tested target locations in the “movement prediction error” condition challenges an assumption implicit in some studies (Held, 1961; Tseng et al., 2007), and it suggests that actual-to-expected reafference discrepancies, when they are within the range of perceptual uncertainty, are insufficient to induce short-term adaptation on their own. This finding contrasts with those of Diedrichsen et al. (2005) and Tseng et al. (2007). Like the present study, these earlier studies involved a pointing experiment toward a visual target. Errors were introduced by random rotation of the visual reafferences of the hand, which is comparable to prism-displaced vision. When they examined the influence of behavioral correction on the next trial, the authors found strong and rapid adaptation effects, as assessed using a state-space model of trial-by-trial adaptation. They concluded that the rotation of the visual reafferences of the pointing movement induced adaptation because it involved a change in the predicted visual reafferences (based on a forward internal model). However, it is important to note that, in these studies, the prediction error was not the only driving the error signal, because hand-to-target feedback error signals were available throughout the movement and at its end.

Mazzoni and Krakauer (2006) demonstrated that if subjects are provided with an intentional strategy to counter a 45° visuomotor rotation, they are able to successfully apply the strategy at first, but then show a gradual drift away from the target. They used an aiming target as a cue to indicate the way to efficiently reach the goal target to counteract the visuomotor rotation. The error signal that drove the implicit adaptation was likely the retinal error signal derived from the “aiming target” (cue)-to-cursor feedback. Moreover, Taylor and Ivry (2011) further investigated the Mazzoni and Krakauer (2006) paradigm. They used three conditions: one with the aiming target permanently available, another one with the aiming target available briefly before movement onset, and a last one with a brief aiming target every two trials. In all three cases they observed a significant aftereffect. When they removed visual markers (in fact once over two successive trials) that provided external landmarks, the degree of drift was sharply attenuated. A brief flashed aiming target either systematic or once over two trials involves a visual short-term memory feedback error, although it reduces its saliency. These results are consistent with our hypothesis that the feedback error plays a key role in the adaptive process. Indeed, the observed drift (and also its correlated aftereffect) was maximum for a permanently lit aiming target, then decreased by half under the flashed aiming target and decreased by a forth for the intermittent flashed aiming target (“no aiming target”). In comparable exposure conditions, Schaefer et al. (2012) observed a limited adaptation to a perturbation applied along task irrelevant dimensions of a movement (amplitude vs. direction).

Magescas and Prablanc (2006) and Laurent et al. (2011) dissociated feedback error from prediction error using a double-step adaptation paradigm, which removed the prediction error by keeping visual reafferences from the hand unaltered. A purely terminal feedback error signal was provided by introducing a target jump, i.e., turning off the target and the vision of the hand at saccade onset. Then, at hand-movement end, the (shifted) target and the hand were made visible again, providing a terminal feedback error signal. In order to keep subjects unaware of the artificially introduced error throughout the exposure paradigm, the target jump was slowly increased. In such an experimental condition, subjects perceived a single target while they were presented a double-step target. The obtained adaptation was large (with an aftereffect of about 30% of the final perturbation) and very robust, without decay during the post-test. In addition, it generalized to a much larger area than the exposed location, ruling out a simple learning phenomenon.

### THE ROLE OF INCREMENTAL EXPOSURE IN ADAPTATION: SELF-ASSIGNMENT OF ERRORS

In Magescas and Prablanc (2006) study, as each increment in target jump was within the natural variability of open-loop visuomotor responses, subjects self-assigned the observed feedback error at movement end, as in the present experiment. Self-assignment of errors by subjects may promote adaptation, irrespective of the source of this error, due, either, to inaccurate goal localization, or to a noisy motor command. A role of self-assignment in motor performance adaptation has been suggested in previous studies (Kording et al., 2007; Kluzik et al., 2008; Schubert and Zee, 2010; White and Diedrichsen, 2010; Schlerf et al., 2013).

Prism adaptation (Michel et al., 2007) and force-field adaptation (Malfait and Ostry, 2004) are highly sensitive to cognitive factors. When errors are naturally attributed to internal causes (due to imprecise definition of the goal, or to erroneous motor command), self-assignment of errors facilitates the development of an unaware adaptive process. Michel et al. (2007) compared adaptation to an unconscious incremental prism exposure from 2 to 10° and to a sudden conscious exposure shift of 10°. They found much larger aftereffects and robustness in the incremental than in the sudden exposure. The association of strong adaptive aftereffects with unconscious perturbations is not limited to prism adaptation. As mentioned above, other motor-adaptation procedures using incremental, sub-threshold steps have also been found to induce large and robust pointing aftereffects (Magescas and Prablanc, 2006; Cameron et al., 2010; Laurent et al., 2011, 2012). In these paradigms, subjects had no knowledge of the perturbation and self-assigned the errors related to inaccurate perception or planning, but they did not attribute the error to a change in the goal.

Differences in the strength of adaptation are likely to be related to the assigned causes of errors. When a subject consciously perceives the perturbation, she/he believes that the observed error is the result of either a change in the external environment or a misrepresentation of his action. It is therefore logical that adaptation, or learning, becomes strongly associated with the context in which it is elicited. The adjustment is then a local rearrangement tied to a particular situation in which the CNS learns a new visuomotor transformation with a narrow spatial (Krakauer and Mazzoni, 2011) or velocity (Kitazawa et al., 1997) adjustment. However, when the perturbation is introduced gradually, the CNS can interpret errors as a result of its own variability, and thus correct some of the basic coordination parameters that underlie the organization of the sensorimotor system. Consistent with this hypothesis, Michel et al. (2007) have suggested that larger and more robust prism-adaptation effects for hemineglect patients than for healthy subjects (Rossetti et al., 1998) is related to the fact that patients do not perceive the disturbance. The mechanisms underlying the alleviation of neglect symptoms by prism adaption have been discussed by Striemer et al. (2008).

Incremental adaptation has obvious cumulative limitations, however. When the incremental perturbation becomes too large, adaptation measured through aftereffect suddenly drops (personal communication). In addition, even if the perturbation is small, the aftereffect decays much quicker when subjects are informed (Cameron et al., 2010) than when they are uninformed of the perturbation.

### SELF-ASSIGNMENT AND PREDICTION

The self-assignment of errors is closely related to the notions of efferent copy and predictive/forward model. The CNS is able to predict sensory reafferences following muscular activation, given the initial state of the body, a copy of muscle commands, and a predictive forward internal model. It can determine, at the end of the action, whether the actual reafferences are compatible with the predicted reafferences. It also can differentiate self-produced from externally produced sensory events (Sperry, 1950; von Holst and Mittelstaedt, 1950; Held, 1961; Bastian, 2006). As motor commands and their translation into movements are necessarily noisy, the predictive model should allow for a margin of error, or confidence interval. Consistent with this idea, some authors have suggested the idea of probabilistic mechanisms in sensorimotor control (van Beers et al., 2002; Kording and Wolpert, 2006; Stevenson et al., 2009; Orbán and Wolpert, 2011). The CNS would assign the error either to the outside world, or to itself, depending on the relative magnitude of the error as compared to the natural noise for the same type of task. In this context, we suggest that an optimal adaptation paradigm requires operating in an intermediate zone, with errors small enough to be self-assigned by the subject, but large enough to induce changes in the inverse model (i.e., in the visual-to-motor transformation), so as to maintain accurate performance and efficient prediction. We propose that this is the case in studies involving prism adaptation using a low deviation (Jakobson and Goodale, 1989), an unaware incremental deviation (Michel et al., 2007), or non-contact Coriolis force-field perturbations acting upon forward-reaching movements during body rotation at constant velocity (Coello and Orliaguet, 1992, 1994; Lackner and Dizio, 1994, 2005; Dizio and Lackner, 1995; Coello et al., 1996). Consistent with this view, Wilke et al. (2013) suggested that, following a rotation of the visual reafferences, the sensorimotor system must be recalibrated using only prediction errors attributed to internal causes.

### UNDERLYING FUNCTIONAL MECHANISMS

Based on the results described above, we propose that two complementary mechanisms exist for visual information processing during prism adaptation, when subjects are unaware of the visuomotor conflict. These mechanisms do not call for any cognitive content or skill learning.

The first mechanism involves accurate, intra-sensory hand-to-target feedback error processing during, or at the end of, movement. When an incremental adaptation is used, the CNS can naturally and iteratively modify visuomotor transformations, i.e., the inverse model. In this context, the adaptive process consists in a series of exponential negative shifts, which updates the inverse model as prism-strength increases.

The second mechanism involves processing of a non-retinal error signal related to the discrepancy between the predicted and seen hand positions – the output of the direct internal model. Small incremental errors do not provide a detectable physical signal, so that modification of the inverse internal model does not occur, at least, in the short term. Under these circumstances, there is no change in the direction of arm movement along the projection axis of the shoulder, as we observed during the exposure phase of the “movement prediction error” condition in the present study. A likely explanation for this outcome is that feedback error has a much lower threshold than prediction error. Prediction error, having a low accuracy, would need feedback error to be updated. Consistent with this view, Popa et al.’s (2012) have suggested that “dual representations of prediction and feedback error within the cerebellum may provide the signals needed to generate sensory prediction errors used to update a forward internal model.”

In contrast with results of the present study, Synofzik et al. (2008) observed adaptation of the inverse model using prediction-error only. They investigated the relationship between the adaptive aftereffect (called motor probe) and the visual prediction of one’s own movement (called perceptual probe), independently from any feedback-error signal, in a paradigm of visual rotation of the hand movement. The visual rotation was introduced in 6° steps, up to 30° clockwise. Subjects were required to perform out-and-back pointing movements in a virtual-reality setup in complete darkness. The authors found nearly 40% adaptation of the perceptual prediction in the direction of the rotated visual reafferences, and a 30% aftereffect in the motor probe test in the direction opposite to the visual rotation, as predicted by coherent adaptation of the inverse and forward models of the arm (Haruno et al., 2001; Flanagan et al., 2003). A possible explanation for the different outcomes of Synofzik et al.’s (2008) study and the present study relates to differences in maximum the stepwise deviation (30° vs. 14°), in addition to protocol differences. A 30° visual rotation of the moving hand in an arbitrary, external coordinates system may be more detectable than a 14° limb deviation in a head-centered reference frame, and thus act as a learning signal, although both distortions were progressively introduced. Synofzik et al. (2008) proposed that a change in the prediction of the visual reafferences produces, in turn, a change in the visual-to-motor transformation. They proposed a quantitative test of the forward-model change following a visuomotor adaptation. Although we did not test it, a change of the forward model in our two “movement prediction error” and “terminal feedback error” conditions is likely, since the final (25 diopters) prism displacement remained undetected by the subjects. As noted by Izawa et al. (2012), some studies suggest the existence of an inverse model that can be learned independently of the forward model, through reinforcement learning (Izawa and Shadmehr, 2011) or repetition (Diedrichsen et al., 2010; Huang et al., 2011; Verstynen and Sabes, 2011). These authors also pointed out that the localization task used by Synofzik et al. (2008; an assay of the forward model) had some issues related to confounding factors: the recorded changes in the perception of the arm position could reflect combined changes in the forward model and in proprioception. Cressman and Henriques (2010) have obtained some evidence consistent with the hypothesis that these changes reflect proprioception recalibration, and are unrelated to an association between motor commands and sensory consequences. Therefore, it is unclear whether changes in the perceived state of the arm after visuomotor adaptation are due to changes in some forward model, or to a form of sensory adaptation. Izawa et al. (2012) and Cressman and Henriques (2010) results, as well as ours, depart from the top down view according to which adaptation of prediction precedes adaptation of control in goal-directed movements as suggested by Flanagan et al. (2003). It is likely that changes that take place in motor commands during adaptation are only partly, and indirectly, driven by changes in forward models.

### ASSOCIATING FEEDBACK AND PREDICTION

Studies using the unaware double-step adaptation paradigm (Magescas and Prablanc, 2006; Cameron et al., 2010; Laurent et al., 2011, 2012) have demonstrated that adaptation can be elicited without adaptation of the predicted visual reafferences, i.e., without a change in the forward model. These findings emphasize the role of visual feedback, but do not negate a role of prediction. Based on the results of our “terminal feedback error” condition, we suggest that optimal conditions for adaptation are obtained when prediction error and a corresponding physical feedback error signal are simultaneously present. The “terminal feedback error” condition also included some predictive component, although not consciously perceived. Advantageous consequences of the association between retinal-feedback and prediction errors for the adaptation of the saccadic system have been suggested recently. In the basic saccadic-adaptation paradigm, the target is moved 20–30% backward during the saccadic suppression period, which opens the retinal feedback loop. The perceptual experience of an overshoot at the end of each saccade produces, after about a hundred trials, a decrease in saccade gain—an image of the inverse model. Collins and Wallman (2012) designed a modified version of the classical saccadic-adaptation paradigm based on the fact that natural saccades have a gain statistically lower than 1, and assuming that the CNS predicts eye movements based on their commands. They compared two conditions with both the same predicted, or unpredicted, retinal error. A much larger level of adaptation was observed for predicted than for unpredicted errors. It suggests that prediction error provides a strong additional adaptive signal.

**FIGURE 4:**
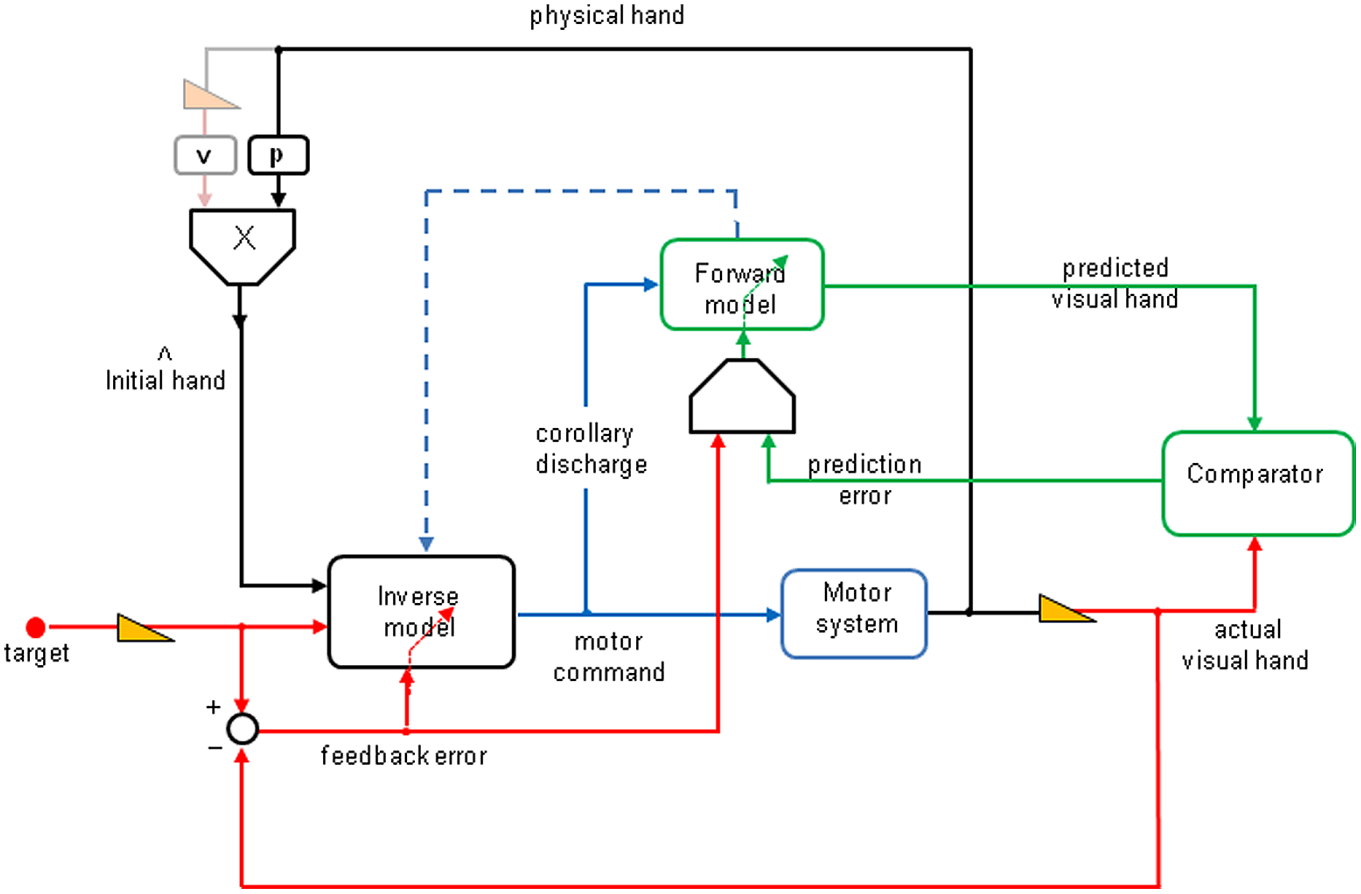
**Functional schema of unaware visuomotor adaptation to lateral prism deviations (derived from Miall and Wolpert, 1996).** The visual target location is laterally shifted using small prism increments, every 10 pointing trials. To produce a reaching movement toward the target, the inverse model uses initial hand estimate and the seen target positions to compute a motor command, which is sent to the motor system. The output of the latter controls the physical position of the hand. The actual prism-displaced hand position (right red arrow) is sent to a comparator. In parallel, the inverse model sends a copy (corollary discharge) to the forward model, the output of which gives a prediction of the hand visual reafferences (upper green arrow) sent to the comparator. The prediction error (lower green arrow) is supposed to iteratively update the forward model, which in turn (blue dotted arrow) updates the inverse model. A new feature, in this schema, relates to the need of combining feedback and prediction error signals to iteratively update the forward model. The visual feedback error signal (red arrow) that is sent to the validation gate (g) has a very low (retinal) detection threshold, whereas the prediction error signal (green) also sent to this validation gate has a high detection threshold, which makes it unreliable alone to induce adaptive updating in the forward model, except for large deviations perceived as resulting from external perturbations. However, for small or moderate prediction errors, the feedback signal allows a disambiguation of the prediction error and allows an adaptive updating of the forward model. The hand-position estimate prior to movement onset is a weighted average of proprioceptive (*p*) and visual (*v*) hand positions; the latter (gray yellow arrow) is absent here.

**Figure 4** shows a very schematic representation of the processes underlying goal-directed adaptation to visually displaced vision of the world and of one’s own body (please refer to the legend for more details). One additional feature to Miall and Wolpert’s (1996) model is suggested by the results of the present study. It highlights the need of combining feedback and prediction error signals to iteratively update the forward model. The visual feedback error signal (red arrow) that is sent to the validation gate (g) has a very low (retinal) detection threshold, whereas the prediction error signal (green) also sent to this validation gate has a high detection threshold, which makes it unreliable alone to induce adaptive updating in the forward model, except for large deviations perceived as resulting from external perturbations. However, for small or moderate prediction errors, the feedback signal allows a disambiguation of the prediction error and allows an adaptive updating of the forward model.

Note that the bias resulted from a weighted average of proprioception and vision of the hand before movement onset (Rossetti et al., 1995) was eliminated here, because vision of the hand prior to the movement was prevented in both the “terminal feedback error” and “movement prediction error” conditions. Whether adaptation of the forward model can automatically induce adaptation of the inverse model, as proposed by Synofzik et al. (2008), cannot be answered based on the present results, because the experiment was not designed to test for changes in the forward model. However, it is likely that, with a much larger prism deviation, the “movement prediction error” condition alone would elicit an adaptation of the forward model, which in turn could induce some adaptation of the inverse model. In the “terminal feedback error” condition, the retinal error signal not only drives the adaptation of the inverse model, it may also refine the prediction error. We suggest that the retinal error signal updates the adaptation of the forward internal model, and strengthens the adaptation of the inverse model.

### BEHAVIORAL COHERENCE OF ADAPTATION PARADIGMS

When considering the behavioral coherence of adaptation procedures for pointing or reaching, unaware double-step paradigms (Magescas and Prablanc, 2006; Cameron et al., 2010) may appear artificial, as they imply an adaptation of the inverse model while the forward model is kept intact, which is not behaviorally coherent. Rotated visual feedback paradigms (Prablanc et al., 1975; Diedrichsen et al., 2005; Tseng et al., 2007) raise another issue with respect to behavioral coherence because, although they allow a coherent modification of the forward and inverse models, they disrupt visual-somatosensory consistency–the object can be reached visually, but not physically. In order to preserve functional coherence, the vision of the hand and the image of the object both have to be rotated. Such a configuration, allowing behavioral and functional coherence of the adaptive process, may be realized in a 3D virtual reality environment, using cyberglove recording and realistic visual feedback; it would mimic prism-displaced visuomotor adaptation, preserving tactile and force feedback.

In addition, most rotated visual feedback adaptation paradigms are hand-centered at an arbitrary point, unrelated to the body or head axis, and their degree of generalization is very narrow (Krakauer et al., 2000; Tanaka et al., 2009). However, because manipulanda rotating feedback paradigms make it easy to manipulate visual and force perturbations, they have made it possible to successfully investigate short-term memory of procedural or skill learning (Miall et al., 2004). Finally, it is worth pointing out that even the most natural adaptation paradigms involve intersensory discrepancies between visual and somatosensory signals. In the present study, we tried to reduce, if not eliminate, such discrepancies. For long-term exposure, behavioral adaptation to prism-displaced vision involves, not only, a change of the inverse and forward models, but also, a series of sensory adaptations (visual and proprioceptive) along the sensorimotor chain: eye, head, and limb (Harris, 1963; Craske, 1967; Redding et al., 2005; Hatada et al., 2006a,b).

### NEURAL CORRELATES OF ADAPTATION

The role of the cerebellum in prism adaptation has been intensively investigated in both primates (Baizer and Glickstein, 1974, 1994; Baizer et al., 1999) and humans (Weiner et al., 1983; Block and Bastian, 2012). It has been suggested that cerebellar influences are involved in a larger network, which includes the posterior parietal cortex (PPC; Clower et al., 1996; Newport et al., 2006; Luauté et al., 2009; Chapman et al., 2011), as well as the ventral premotor cortex–a major target of cerebellar output (Kurata and Hoshi, 1999). In anatomical studies in monkey, Prevosto et al. (2010) have identified projections from the cerebellar nuclei and cortex to the medial intraparietal area and the ventral premotor cortex, consistent with an involvement of ventral premotor cortex in prism adaptation. In a recent fMRI study of prism adaptation, Küper et al. (2013) identified an ipsilateral activation associated with the early strategic motor control responses within the posterior cerebellar cortex and the dentate nucleus. However, Pisella et al. (2004) showed that adaptation to a 15° prism displacement was still possible with a bilateral PPC lesion, and they suggested that the PPC was primarily associated with the strategic component of adaptation. Robertson and Miall (1999) compared the differential involvement of the cerebellum in gradual or sudden adaptation to rotated visual feedback, the former being more altered than the latter by inactivation of the dentate nucleus. Concerning the selective involvement of the cerebellum in inverse and forward models in the context of progressive visual rotation paradigms, Synofzik et al. (2008) tested cerebellar patients and healthy subjects using the same rotation paradigm. The former exhibited 20% adaptation of the perceptual prediction, with no significant aftereffect—suggesting no significant adaptation of the inverse model. By contrast with Synofzik et al. (2008) and Izawa et al. (2012) found similar aftereffects for cerebellar patients and control subjects following exposure to a 30° visual rotation in 5° steps, indicating that the inverse model was adapted. However, unlike for control subjects, adaptation of the inverse model was not associated with a change in motor-response prediction. Therefore, while the role of the cerebellum in prism adaptation is relatively well established, it is not clear whether the forward and the inverse models (i.e., the predictive and feedback adaptive processes) depend upon distinct regions of the cerebellum.

## CONCLUSION

The results of this study provided evidence that short-term visuomotor adaptation induced using gradual, sub-threshold prism displacement requires some feedback from the hand-to-target signal. The processing of successive errors led to a gradual reduction of errors in the absence of strategic behavior. These findings moderate previous interpretations, according to which discrepancies between actual and predicted movement reafferences are the main, and perhaps the only, source of visuomotor adaptation induced by exposure to prisms that shift the visual field or to rotated visual coordinates. Our finding, that retinal hand-to-goal feedback is necessary for updating the prediction of reafferences when a visual perturbation is introduced gradually and cognitive factors are eliminated or strongly attenuated, suggests a reversal of the causality between prediction and feedback processes in unaware visuomotor adaptation. Although predictive behavior remains one of the cornerstones of sensorimotor organization, these results support the view that a high level of visuomotor performance depends upon continuous updating of action predictions through sensory-feedback processing. In the present study, any initial (prior to movement) conflicts between proprioception and vision were deliberately removed in order to facilitate comparisons with visual-rotation paradigms. The influence of intersensory conflicts during movement was further reduced through progressive, and limited, prism displacements. An important goal for future studies and models of long-term adaptation is to determine the contribution of motor adaptation and its visual prediction, and of the many sensory processes, including visual, oculomotor, neck, and limb proprioceptive adaptation, which together contribute to smooth, accurate and context-independent adaptive behavior.

## Conflict of Interest Statement

The authors declare that the research was conducted in the absence of any commercial or financial relationships that could be construed as a potential conflict of interest.
